# RETINAL FLECKS IN STARGARDT DISEASE REVEAL CHARACTERISTIC FLUORESCENCE LIFETIME TRANSITION OVER TIME

**DOI:** 10.1097/IAE.0000000000002519

**Published:** 2019-04-10

**Authors:** Yasmin Solberg, Chantal Dysli, Pascal Escher, Lisa Berger, Sebastian Wolf, Martin S. Zinkernagel

**Affiliations:** * Department of Ophthalmology, Inselspital, Bern University Hospital, Bern, Switzerland; and; † Department of BioMedical Research, University of Bern, Bern, Switzerland.

**Keywords:** fluorescence lifetimes, fundus autofluorescence, ophthalmic imaging, Stargardt disease, macular dystrophy

## Abstract

**Purpose:**

Stargardt disease is the most common inherited juvenile macular dystrophy and is characterized by yellowish flecks across the posterior pole. The purpose of this study was to investigate fluorescence lifetime changes of retinal flecks over time using fluorescence lifetime imaging ophthalmoscopy.

**Methods:**

Longitudinal fluorescence lifetime data of 12 patients with Stargardt disease (mean age ± SEM, 42.25 ± 2.1 years; range, 28–58 years) were acquired using a fluorescence lifetime imaging ophthalmoscope based on a Heidelberg Engineering Spectralis system. Retinal autofluorescence was excited with a 470-nm laser. The emitted fluorescence was detected in two wavelength channels: a short spectral channel (498–560 nm) and a long spectral channel (560–720 nm). The mean retinal autofluorescence lifetimes were calculated and further analyzed with corresponding color fundus images, autofluorescence intensity images, and spectral domain optical coherence tomography. Patients were classified into three subtypes.

**Results:**

All patients with Stargardt disease displayed characteristic autofluorescence lifetime patterns. Mean fluorescence lifetime values within areas of yellow flecks were significantly prolonged (long spectral channel 484 ps) compared with the surrounding tissue (long spectral channel 297 ps). In 91.6% of the eyes, flecks with short fluorescence lifetimes (long spectral channel 255 ps) were identified. Short lifetime flecks progressed to flecks with characteristic long lifetimes in 75.1% of eyes within a mean interval of 29.2 months (range 3–45 months). Between baseline and follow-up, the rate of newly developed short lifetime flecks (number/per year) based on subtypes was 2.62 in Group 1, 1.43 in Group 2, and 0.81 in Group 3.

**Conclusion:**

Recent onset flecks in Stargardt disease display short fluorescence lifetimes and convert into longer fluorescence lifetime flecks over time. This transition may represent a change in the composition of retinal deposits with accumulation of lipofuscin and retinoid by-products from the visual cycle. With emerging treatment options, these findings may prove useful to monitor disease progression and therapeutic effects.

## Abstract 2

In Stargardt disease, retinal fluorescence lifetimes can be used to analyze hyperfluorescent flecks and hypofluorescent atrophic lesions. This study establishes that autofluorescence lifetime demonstrates explicit, reproducible patterns and confirms the diagnostic value of fluorescence lifetime imaging ophthalmoscopy as a noninvasive imaging modality.

Supplemental Digital Content is Available in the Text.

Stargardt disease (STGD) is the most common inherited macular dystrophy. It is a form of macular degeneration initially described by Stargardt in 1909.^1^ Stargardt disease classically presents during the first two decades of life and is characterized by a progressive bilateral loss of central vision. Morphological features include central macular atrophy and well-defined yellowish deposits visible on the posterior pole around the macular, known as “flecks.”^1,2^ Histologically, flecks are located at the level of the retinal pigment epithelium (RPE).^3,4^

Over the course of the disease, initially well-defined flecks progress outward from the central macula in a centrifugal pattern. Flecks then fade, leaving poorly demarcated yellow lesions and residual atrophy.^2,5,6^ Thus, at advanced stages of the disease, a progressive bilateral atrophy of the RPE, photoreceptors, and choroidal vasculature can be found.^7^ The severity of fundus abnormalities seen on ophthalmoscopy is often not directly associated with the visual acuity. However, the overall prognosis of STGD is poor, and visual acuity often deteriorates to 20/200 or less.^8,9^

Stargardt disease is predominately inherited in an autosomal recessive trait, and most cases are caused by pathogenic variants in the retinal-specific ATP-binding cassette transporter gene *ABCA4*, located on chromosome 1p13 (STGD1; OMIM #248200).^10–12^ *ABCA4* expression is predominantly localized to the rim of photoreceptor outer segments and at lower levels in the retinal pigment epithelium.^13^ The ABCA4 transporter flips the N-retinylidene-phosphatidylethanolamine (N-ret-PE) that forms in the visual cycle by reversible combination of toxic retinaldehyde with phosphatidylethanolamine (PE), from the luminal to cytoplasmic surface of photoreceptor outer disc membranes. In the absence of ABCA4 flippase activity, clearance of retinaldehyde is delayed, favoring a secondary condensation of N-ret-PE with another retinaldehyde to form phospholipid-conjugated bisretinoids, v.g. dihydro-N-retinylidene-N-retinyl-phosphatidylethanolamine (A2PE-H2), or its oxidized form (A2PE).^12,14^ Characteristic phenotypic features, such as pisciform flecks and atrophy, are then believed to develop as a result of the accumulation of these retinoid by-products or lipofuscins in the RPE.^10^ In rare cases, STGD is caused by dominantly inherited variants in *ELOVL4* encoding the elongation of very long chain fatty acids protein 4 (STGD3; OMIM #600110) and in *PROM1* encoding the rim protein prominin-1 (STGD4; OMIM #603786).

Several noninvasive imaging techniques have been established in the diagnosis of STGD, and for monitoring disease progression. Spectral domain optical coherence tomography (OCT) is a recognized imaging tool that provides morphologic information by visualizing the retinal architecture. Spectral domain OCT may demonstrate thinning of retinal layers and a loss of photoreceptor segment layers.^15,16^

Fundus autofluorescence (FAF) intensity imaging is an established imaging technique providing information on the retinal status by enabling the visualization of hyperautofluorescent material such as bisretinoid components, such as lipofuscin, originating from the RPE.^17^ In early stage of STGD, FAF imaging reveals hyperautofluorescent lesions consistent with visible flecks on fundus examination. In later stages, hypoautofluorescent areas appear, consistent with RPE atrophy and photoreceptor loss.^6^

Despite substantial research efforts in the past decades, there are no approved therapeutic treatments for STGD. The disease causes significant morbidity with psychological and economic implications. However, promising new options, including gene replacement therapy, stem cell transplantation, and deuterated vitamin A, are emerging and are currently being reviewed in clinical trials.^18,19^ A further challenge researchers face in treating STGD is the lack of a highly sensitive measurement tool to monitor short-term changes in disease progression.^19^ This will be vital to assess effects of future therapies accurately.

Fluorescence lifetime imaging ophthalmoscopy (FLIO) measures lifetimes of endogenous retinal fluorophores after excitation using a picosecond-pulsed blue laser light. A previous report from our group demonstrated characteristic fluorescence lifetime changes across the posterior pole in patients with STGD. In this previous study, retinal flecks with a broad range of autofluorescence lifetimes were identified, and it was speculated that short lifetime flecks represent recent lesions, whereas flecks with longer lifetimes represent older lesions.^20^ The aim of this study was to investigate the dynamics of fluorescence lifetimes within retinal flecks over time.

## Methods

Twelve patients with a clinical diagnosis of STGD were included in the study. The patients were consecutively recruited at the University Hospital in Bern, Switzerland. The diagnosis of STGD was established by findings on clinical examination and ancillary studies including color fundus images, FAF intensity images, and electroretinographic results.^21^ Moreover, 11 of 12 patients with STGD included had a molecular diagnosis of STGD (see **Table 1, Supplemental Digital Content 1**, http://links.lww.com/IAE/A990).

All patients had a baseline and a follow-up examination. At each visit, best-corrected visual acuity (BCVA; according to Early Treatment Diabetic Retinopathy Study [ETDRS] letters^22^) was measured in all patients, and a general dilated ophthalmologic examination was performed. Maximal pupil dilation was achieved using tropicamide 0.5% and phenylephrine HCl 2.5%. Fundus color images (Zeiss FF 450plus; Zeiss, Oberkochen, Germany), OCT scans of the macula (Heidelberg Spectralis HRA + OCT; Heidelberg Engineering, Heidelberg, Germany), and fluorescence lifetime images were obtained of both eyes.

Patients with STGD were assigned to phenotypic subtypes at baseline and follow-up. Based on the clinical appearance of the color fundus and FAF images, patients were assigned to one of three groups:

1. Group I: flecks and atrophy are confined to the central macular area.
2. Group II: flecks are scattered, can spread nasally to the optic disk, and/or exceed the vascular arcades.
3. Group III: flecks are ill-defined “resorbed” lesions, and extensive RPE atrophy is visible.^23^

The study was conducted at the Department of Ophthalmology at the University Hospital in Bern, Switzerland, with the approval of the local ethics committee and in accordance with the Declaration of Helsinki. All participants provided written informed consent before study entry. This study is registered at ClinicalTrials.gov as “Measurement of Retinal Autofluorescence with a Fluorescence Lifetime Imaging Ophthalmoscope (FLIO Group),” with the identifier number NCT01981148.

### Fluorescence Lifetime Imaging Ophthalmoscope

Fluorescence lifetime imaging ophthalmoscope, based on an HRA Spectralis system (Heidelberg Engineering), was used to obtain retinal fluorescence lifetime data. The principles and details of FLIO have been described elsewhere.^22,24^

In summary, the technique is based on the excitation of retinal autofluorescence using a 470-nm pulsed laser at 80-MHz repetition rate. Highly sensitive hybrid photon-counting detectors (HPM-100–40; Becker & Hickl, Berlin, Germany) were used for the registration of emitted fluorescence light by time-correlated single-photon counting modules (SPC-150; Becker & Hickl). Emitted fluorescence photons were measured in two separate wavelength spectrums: a short spectral channel (SSC: 498–560 nm) and a long spectral channel (LSC: 560–720 nm). In both wavelength channels, at least 1,000 photons per pixel were obtained, which requires scan duration of 90 seconds per eye approximately in patients with STGD. During data accumulation, an eye movement tracking system with a high-contrast confocal infrared image ensures the correct location of each detected photon within a field of 256 × 256 pixels.

### Fluorescence Lifetime Data Analysis

Fluorescence lifetime data collection resulted in a decay curve which was biexponentially approximated using SPCImage software version 4.6 (Becker & Hickl). The chi-square value evaluated appropriateness of the exponential fit. The resulting short and long lifetime components (T1 and T2) along with their respective relative amplitudes α1 and α2 were used to calculate the mean fluorescence lifetime *Tm*, which signifies the amplitude weighted mean fluorescence lifetime.

The mean fluorescence lifetimes were analyzed using “FLIO reader” software (ARTORG Center for Biomedical Engineering Research, University of Bern, Switzerland). To spatially quantify retinal autofluorescence lifetimes, a standard ETDRS grid was used with following circle diameters: 1 mm for the central area, 3 mm for the inner ring, and 6 mm for the outer ring. Smaller region of interests with diameters of 0.16 mm and 0.5 mm were used to analyze specific areas of interest, for example, flecks. Fluorescence lifetimes within flecks were analyzed by averaging three regions of interest.

### Statistical Data Analysis

Fluorescence lifetime values for the SSC and the LSC were analyzed separately. All data were presented as mean ± SEM. Statistical analysis was performed using GraphPad (Prism 6; GraphPad Software, Inc, La Jolla, CA). To compare the results, the Mann–Whitney test with a confidence interval of 95% was used. *P* values <0.05 were considered as statistically significant. A stepwise forward regression analysis was performed using SigmaPlot Version 12.3 (Systat Software, Inc, San Jose, CA).

## Results

Twenty-four eyes of 12 patients with a clinical diagnosis of STGD were included in this study (53.8% female). Patient characteristics are shown in Table 1. The mean age ± SEM at baseline was 42.25 ± 2.1 years (range, 26–56). All participants were white, phakic with clear media, and had no concomitant ophthalmic diseases. The examined patients displayed various stages of hyperautofluorescent flecks and hypoautofluorescent lesions, consistent with RPE changes and atrophy. All patients had a baseline and at least one follow-up examination with a mean interval of 29.2 months (range, 3–45 months). All patients developed new flecks during the clinical follow-up period.

**Table 1.**
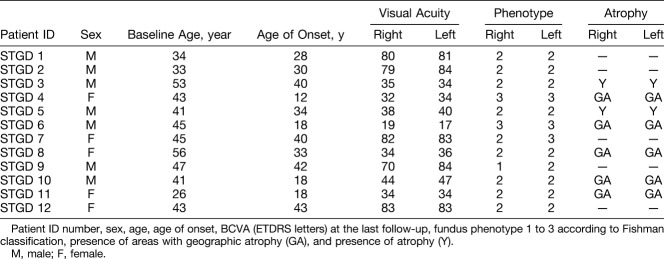
Baseline Patient Characteristics

**Fig. 1.**
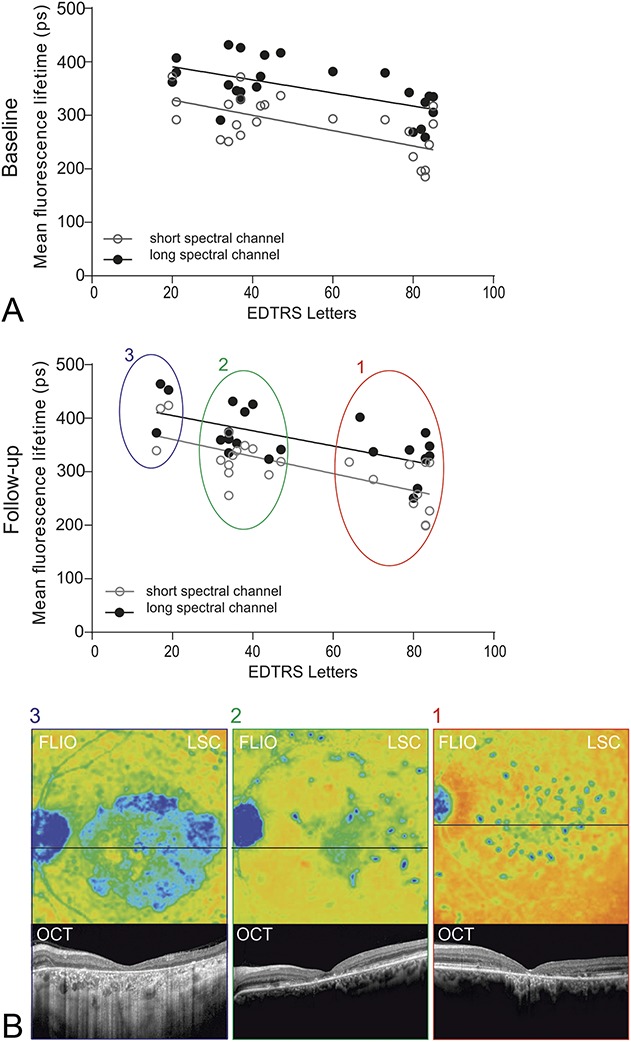
Correlation of BCVA (ETDRS letters) with mean fluorescence lifetime (ps) within the central ETDRS subfield of the short (white circle, SSC, 498–560 nm) and the long (black circle, LSC) spectral channels at (**A**) baseline (SSC: r^2^ = 0.35, *P* = 0.002; LSC: r^2^ = 0.32, *P* = 0.004) and (**B**) follow-up (SSC: r^2^ = 0.51, *P* < 0.0001; LSC: r^2^ = 0.45, *P* = 0.0001). Representative fluorescence lifetime (FLIO, LSC) images of three identified groups and correlating OCT scan of the indicated lines in the FLIO images are shown below.

Mean age at the initial diagnosis of STGD was 29.7 ± 3.1 years (range 12–43). The mean disease duration at the time of the first and the last FLIO measurement was 12.1 ± 3.1 years (range, 0.08–31), and 14.6 ± 3.24 years (range, 5–30), respectively. The mean BCVA at baseline and at follow-up was 53.6 ± 4.9 (range 17–84) and 51.2 ± 4.8 (range 16–83) ETDRS letters, respectively, with a mean EDTRS visual acuity reduction during the follow-up interval of 2.4 letters. There was a significant correlation between the BCVA and the mean fluorescence lifetime of the central ETDRS subfield in both spectral channels when analyzing the data of all study eyes at baseline (SSC: r^2^ = 0.35, *P* = 0.002; LSC: r^2^ = 0.32, *P* = 0.004) and follow-up (SSC: r^2^ = 0.51, *P* < 0.0001; LSC: r^2^ = 0.45, *P* = 0.0001) (Figure 1). A central atrophic lesion was found in 33.3% patients (10 eyes), with a mean size of 7.35 mm^2^ (range 0.48–20.3 mm^2^) at baseline, and 10.6 mm^2^ (range 1.69–28.7 mm^2^) at follow-up. The mean progression rate of the atrophic area was 1.7 mm^2^ per year.

### Autofluorescence Lifetimes in Patients With Stargardt Disease

The white-yellow STGD flecks visible on fundus examination correlated with hyperautofluorescent lesions in FAF intensity images and hyperreflective changes seen in OCT. In FLIO, flecks exhibited both short and long fluorescence lifetimes, when compared with the surrounding retina, and were represented as red and blue lesions in color-coded images with a range between 200 ps (red) and 600 ps (blue).

**Fig. 2.**
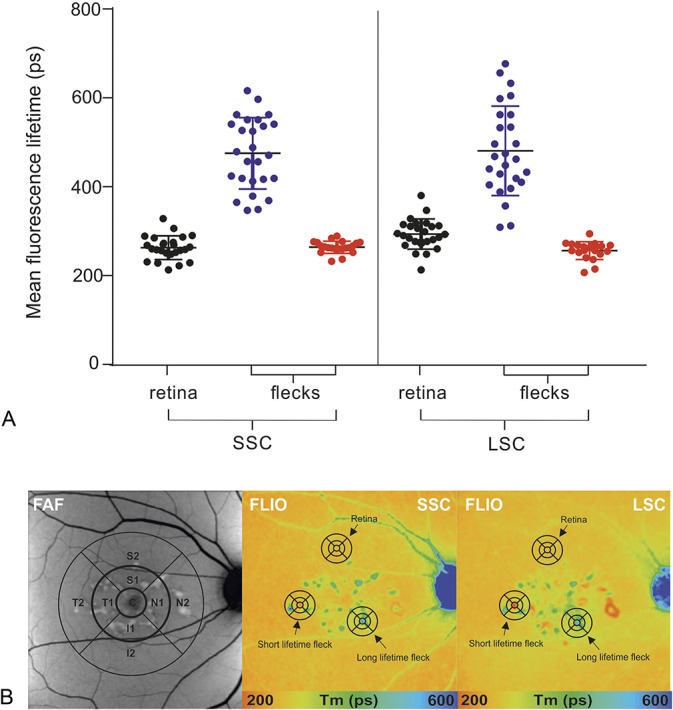
**A.** Quantitative analysis of mean retinal autofluorescence lifetime values in the SSC and LSC in patients with STGD. Following areas were analyzed: surrounding retinal tissue (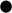), flecks with long (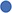) and short (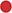) fluorescence lifetimes. **B.** A small region of interest (circle diameter: 0.16 mm) was used to investigate mean retinal autofluorescence lifetimes within short lifetime flecks, long lifetime flecks, and the surrounding retina. Areas of interest represent a mean value of three individual measurement locations. Fluorescence lifetime data of both spectral channels were analyzed separately.

Most flecks displayed long (blue) fluorescence lifetimes (SSC: 4,715 ± 17 ps; LSC: 484 ± 21 ps). However, in 91.7% (n = 22) of eyes, a smaller number of flecks with shorter lifetimes, color-coded in red (SSC: 265 ± 2 ps; LSC: 255 ± 4 ps), were identified (Figure 2).

Compared with the surrounding retina (SSC: 270 ± 6 ps; LSC: 297 ± 7 ps), Ƭm was significantly prolonged in long fluorescence lifetime flecks by +74% in the SSC and +63% in the LCS (both *P* < 0.0001). While in short fluorescence lifetime flecks, Ƭm was shortened by −2% in the SSC and −16% in the LSC (SSC: *P* = 0.21, LSC: *P* < 0.0001) compared with the surrounding retina.

Fluorescence lifetime imaging ophthalmoscopy was used to examine the progression of the disease in our study eyes. Long fluorescence lifetime flecks presented as isolated deposits or as several flecks merging with each other in a meshwork pattern. As expected, fleck formation progressed centrifugal outward from the central macular area as the disease advanced. On follow-up, new flecks developed in all patients; however, the rate and extent varied between patients.

#### Short fluorescence lifetime flecks

**Fig. 3.**
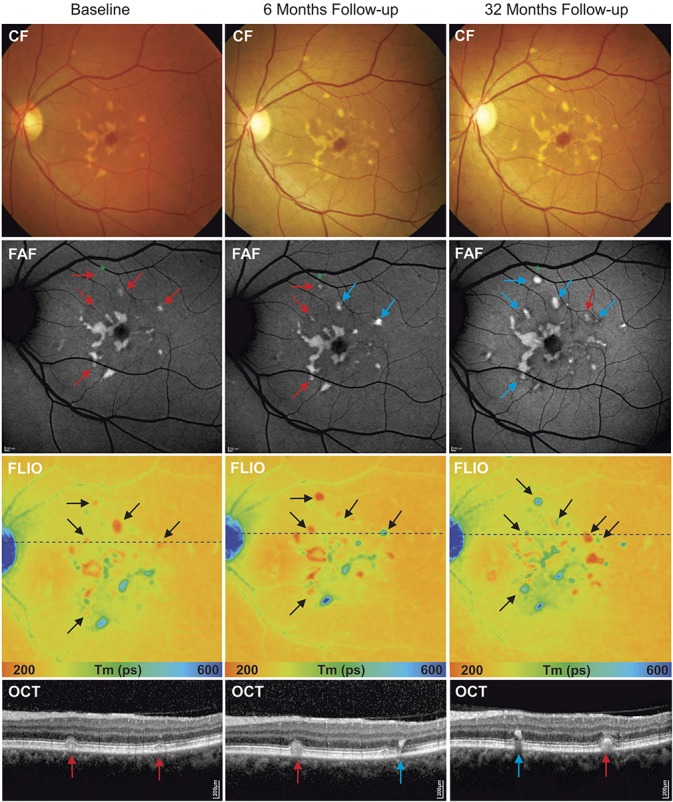
Disease progression within 6- and 32-month follow-up (Patient STGD 9). Color fundus (CF), FAF intensity, and fluorescence lifetime (FLIO, LSC) images. Correlating OCT scan of the indicated lines in the FLIO images are shown below. From baseline (left) to 6-month follow-up (middle), to 32-month follow-up (right), clear disease progression is visible with transition of flecks with short fluorescence lifetimes (red arrows and corresponding black arrows in FLIO) to flecks with long fluorescence lifetimes (blue arrows and corresponding black arrows in FLIO) and appearance of new hyperfluorescent flecks. *Indicates a fleck with short fluorescence lifetime initially not visible on FAF intensity image at baseline, but detectable on follow-up.

A larger number of short fluorescence lifetime flecks were identified in patients at early stages of disease (Group 1 and Group 2), while at later stages, they were only found occasionally. The ratio of short fluorescence lifetime flecks identified at baseline between Group 1 versus Group 3 was 9:5. Quantitative analysis of flecks with short fluorescence lifetimes at two points in time (baseline and last follow-up) revealed that new flecks visible in FLIO developed at a rate of 2.62/year in Group 1, 1.43/year in Group 2, and 0.81/year in Group 3. Short lifetime lesion progressed to flecks with characteristic long lifetimes in 75.1% (n = 22) within 29.2 months. In the transition phase, longer lifetimes initiate in the center of the flecks, and radiate outward with time. Borders of short lifetime flecks shifted to long lifetimes last (Figure 3).

#### Long fluorescence lifetime flecks

**Fig. 4.**
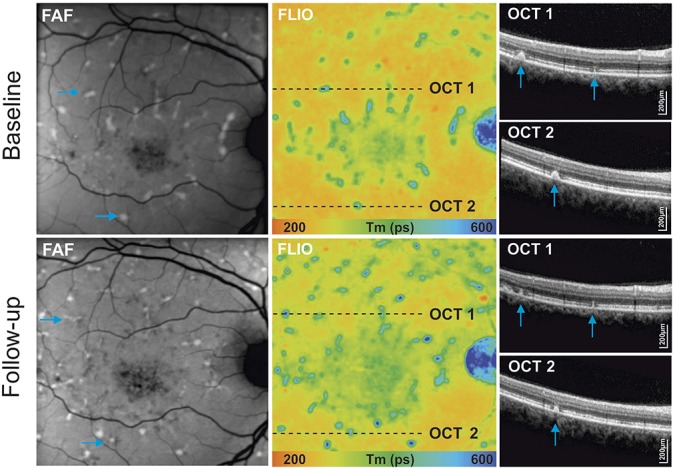
Analysis of disease progression of hyperfluorescent flecks within 44-month follow-up: autofluorescence intensity image (FAF), and fluorescence lifetime (FLIO, LSC) with selected hyperfluorescent flecks (blue arrows) and corresponding OCT scans. Follow-up examinations show a clear progression of flecks in different imaging modalities, with changes in intensities and hyperreflective material deposits on OCT.

In general, there was a predominance of long fluorescence lifetime flecks in all stages of STGD in our cohort. On analysis of long lifetime flecks, borders were identified to feature shorter mean fluorescence lifetime values than the center of the fleck (SSC: 446.5 ± 17 ps, vs. 522.6 ± 20, *P* < 0.0001) (LSC: 462.7 ± 14 ps, vs. 548.3 ± 21 ps, *P* < 0.0001). Borders demonstrated progression from sharp defined edges to a more diffuse pattern (Figure 4).

### Correlation of Fluorescence Lifetime Data With Optical Coherence Tomography Findings

**Fig. 5.**
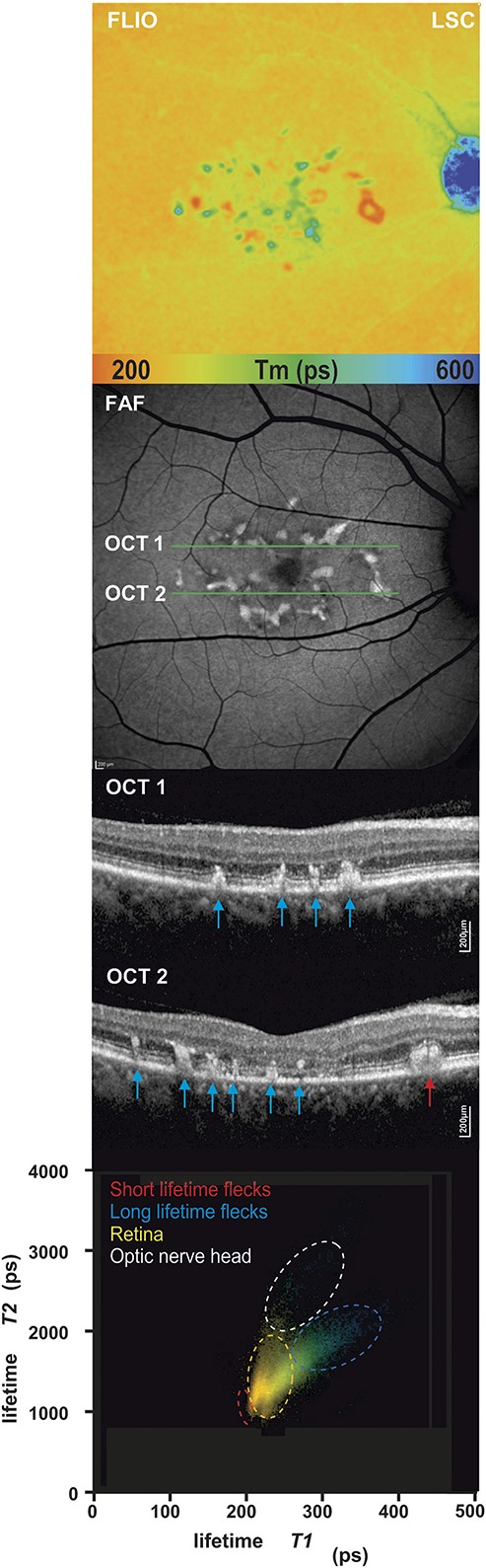
A 2D analysis of short and long fluorescence lifetime components T1 and T2. Distribution histograms (bottom) are shown of a patient with STGD with short and long fluorescence lifetime flecks. Specific areas are highlighted according to the lifetime distribution clouds: surrounding retina, short fluorescence lifetime flecks, long lifetime flecks, and optic nerve head. Corresponding fluorescence lifetime image (FLIO, LSC), FAF intensity, and OCT scan.

Colocalization of flecks in FLIO with OCT displayed hyperreflective material of variable size and shape. At the level of the flecks, OCT bands displayed convex, pyramidal-shaped, or ill-defined deposits, interrupting the photoreceptor ellipsoid zone (EZ) and/or external limiting membrane bands. Some deposits displayed hyperreflective foci extending into the outer nuclear layer, with a remaining connection to the RPE. Depending on the location of hyperreflective deposits in OCT, FLIO images displayed characteristic lifetime patterns. Long fluorescence lifetime flecks generally demonstrated intraretinal migration of deposits of varying extents (Figures 4 and 5), while short fluorescence lifetime flecks usually presented as dome-shaped deposits located within the outer retina at the level of the RPE, with displacement or interruption of the photoreceptor segments (Figure 3).

### Individual Fluorescence Lifetime Components

As described in the method section, *Tm* is composed of the separate lifetimes components T1 (short portion) and T2 (long portion), along with their corresponding amplitudes or intensities (α1 and α2). We analyzed these individual fluorescence lifetime components independently using 2D histograms to observe their effect on *Tm* as described in previous publications.^22,24^

Our data revealed that both short and long fluorescence lifetime flecks can be isolated from the surrounding retina. Short lifetime flecks characteristically showed a shorter T1 and T2 component in comparison to the surrounding retina and the long lifetime flecks (Figure 5).

### Regression Analysis

The influence of the independent variables patient's age, disease duration, and Fishman score was analyzed against the dependent variables mean fluorescence lifetime (*Tm*) in the SSC and the LSC. When analyzing mean fluorescence lifetime values within the central ETDRS grid area, the disease duration significantly contributed to the ability of the equation to predict lifetimes in the short and long wavelength spectrum (SSC: *P* = 0.02 and LSC: *P* = 0.04). When analyzing unaffected surrounding retina, only age significantly correlated with longer mean fluorescence lifetimes within the SSC (*P* = 0.006).

## Discussion

In this prospective study, we investigated retinal fluorescence lifetimes in 24 eyes of patients with STGD using fundus autofluorescence lifetime (FLIO) imaging. Previously described characteristic lifetime patterns^20^ were confirmed. Our data showed that most retinal hyperautofluorescent flecks and deposits displayed longer lifetimes compared with the surrounding retina. However, particularly at early stages of disease (Groups 1 and 2), lesions with very short fluorescence lifetimes were identified. These lesions were more apparent in the LSC (560–720 nm). On correlation to other imaging modalities, we observed that flecks with short fluorescence lifetimes were either not yet visible, or faint and ill-defined on FAF intensity images, while flecks with prolonged lifetimes were clearly identifiable. Interestingly, our follow-up measurements showed that lesions with initially short lifetime values which were faintly visible in FAF intensity measurements progressed to flecks which were clearly visible as hyperautofluorescent flecks and displayed long fluorescence lifetimes. These results are in keeping with our previous study of FLIO in STGD.^20^ In this study, we provide evidence that flecks with short lifetimes occur more frequently at earlier and more active stages of the disease, and may be the result from the buildup of intermediate components of the retinoid cycle.^11^ On the other hand, blue flecks represent a more advanced stage of the lesion, possibly indicating a more severe dysfunction of the underlying retina as by-products of the visual cycle accumulate. Sparrow et al^25^ proposed that in STGD by-products of the visual cycle can buildup in the outer segment of photoreceptor cells.

Histopathological studies suggest that the hyperautofluorescent flecks originate from lipofuscin accumulation in the RPE cells, which in STGD occurs at an accelerated rate in comparison with the normal aging process.^26,27^ Buildup of fluorescent lipofuscin in the RPE is a hallmark of STGD, and the possible disease mechanism due to the dysfunction of the ABCA4 flippase could be the following.^13^ After photoactivation of rhodopsin, all-trans retinal builds up in the photoreceptor outer segments and readily forms N-retinylidene-phosphatidylethanolamine (N-ret-PE) by binding to amine groups of phosphatidylethanolamine (PE). Loss of ABCA4 flippase activity reduces clearance of all-trans retinal and favors its condensation with a second retinaldehyde to form the bisretinoids A2PE. On phagocytosis, A2PE is converted into the major lipofuscin fluorophore A2E (N-retinylidene-N-retinylethanolamine) in the acidic environment of RPE phagosomes.^26–28^ The recent identification of ABCA4 in RPE internal membranes is in further support that accumulation of autofluorescent precursors may contribute to both RPE- and outer photoreceptor segment dysfunction, leading to lipofuscin buildup.^13,29^ These equilibriums possibly contribute to the shorter lifetimes identified in the newly formed deposits in STGD.^20,22^

In addition, other components such as melanin have been suggested to be involved in the disease process. Melanin is concentrated at the fovea in the RPE cells and has been shown to decrease in normal aging.^30^ It has been speculated that lipofuscin buildup in the RPE cells is partly influenced by melanin.^31^

As described in previous clinical studies, our longitudinal FLIO measurements revealed that fleck formation begins centrally in the foveal area, radiating outward as the disease progresses. In addition, new lesions often develop in close proximity to existing flecks, probably due to toxic effects of visual by-products on neighboring RPE cells and photoreceptors.^5^

Despite previous studies, the exact location of flecks remains controversial. On colocalization of the flecks to the OCT bands, we identified a variety of shapes and sizes of hyperfluorescent accumulations at the EZ and/or external limiting membrane bands. However, an anatomical link to the RPE layer was present, suggesting that the lipofuscin deposits begins in the RPE and then migrates toward the inner retinal layers. As previously observed by Voigt et al,^32^ we hypothesize that the different forms of deposits observed in the OCT bands represent different phases in the natural history of fleck progression. Although flecks with varied fluorescence intensities were identified, we could not detect a correlation to the interruption of EZ, external limiting membrane, or outer nuclear layer bands. On analysis of OCT sections, we observed that at earlier stages of the disease, the hyperreflective material was restricted to the area underneath the EZ, whereas at later stages, lesions with longer lifetimes tended to progress through the EZ into the outer retinal layers.

A useful tool to investigate the characteristics and progression of STGD is the 2D histogram analysis. Information from endogenous fluorophores can be obtained by analyzing the individual short (T1) and long (T2) decay components, which contribute to the mean fluorescence lifetime (*Tm*). As different fluorophores have distinct lifetime patterns, specific retinal structures can be demarcated. This technique can be used to identify short and long decay times in separate clusters (Figure 5). Furthermore, analysis of long lifetime flecks revealed that the borders display shorter lifetimes than the center of a fleck. These findings are in keeping with the observation that short lifetime flecks progress to long lifetime flecks from the center outward. This suggests that the short fluorescence lifetime flecks are composed of slightly different fluorophore components than the long lifetime flecks. Sparrow et al^33^ identified an increased signal within flecks using short-wavelength FAF. They purposed that early on in STGD, the RPE cell layer is altered, and that the enhanced short-wavelength FAF signal could arise secondary to RPE atrophy and augmented lipofuscin accumulation. These findings may provide some insight into the possible sequence of retinal changes in STGD.

There are some limitations to this study. Primarily, in one patient, the diagnosis of STGD was largely based on clinical assessment, and in one patient, an autosomal dominant inheritance trait was identified. Patients displayed a variable degree of disease severity from early or childhood-onset to late-onset of STGD. We also acknowledge that the data provided in this report showed a cohort of patients with an atypical presentation including an older age of onset of symptoms and a higher progression rate of atrophic lesions in comparison to the ProgStar cohort.^34,35^ Furthermore, a larger sample size would have provided more information. However, despite the small cohort, the findings of conversion of retinal flecks were consistent between patients. Concerns about the long-term effects and the relationship between short-wavelength light and the generation of toxic products have been raised, especially patients with STGD. However, the light exposure of the FLIO device is well below the limits recommended by the ANSI standard for safe use of lasers.^36^

## Conclusions

In STGD, retinal fluorescence lifetimes can be used to analyze hyperfluorescent flecks. Fluorescence lifetime imaging ophthalmoscopy has the potential of becoming a monitoring tool in retinal conditions by illustrating metabolic malfunctions and providing further information about the composition of endogenous retinal fluorophores. This study establishes that fundus autofluorescence lifetimes demonstrate explicit, reproducible patterns in STGD, and confirms the diagnostic value of FLIO as a non-invasive imaging modality.

Using FLIO, we present a method of monitoring and quantifying disease progression in patients with STGD. Patterns of disease progression, when quantified, could be useful in the development of new outcome measurements for clinical trials testing novel therapies for STGD. Since these changing autofluorescence lifetime patterns may reflect intracellular events in RPE cells, they may be helpful in gauging the biological effect of potential therapies and in interpreting treatment effects.

## Supplementary Material

SUPPLEMENTARY MATERIAL
