# Supplementary material for: RETINAL FLECKS IN STARGARDT DISEASE REVEAL CHARACTERISTIC FLUORESCENCE LIFETIME TRANSITION OVER TIME
Source: Retina. 2019 Apr 10;39(5):879–88. doi: 10.1097/IAE.0000000000002519 (PMC6510322; doi:10.1097/IAE.0000000000002519)
Supplement: SUPPLEMENTARY MATERIAL [file retina-39-879-s001.docx]

**Supplemental Digital Content**

**INTRAVITREAL AFLIBERCEPT FOR PATIENTS WITH DIABETIC MACULAR EDEMA FROM JAPAN**

HIROKO TERASAKI, KUNIHIKO SHIRAKI, MASAHITO OHJI, CAROLA METZIG, THOMAS SCHMELTER, OLIVER ZEITZ, OLAF SOWADE, MASATO KOBAYASHI, ROBERT VITTI AND ALYSON BERLINER, AND FUMIO SHIRAGA

**1. Exclusion Criteria**

Patients who met any of the following criteria were excluded from the study:

1. Ocular conditions with a poorer prognosis in the fellow eye than in the study eye
2. History of vitreoretinal surgery and/or including scleral buckling in the study eye
3. Laser photocoagulation (panretinal or macular) in the study eye within 30 days before Day 1
4. Previous use of intraocular or periocular corticosteroids in the study eye within 120 days before Day 1
5. Previous treatment with antiangiogenic drugs (pegaptanib sodium, bevacizumab, ranibizumab, etc) in either eye within 90 days before Day 1
6. Active proliferative diabetic retinopathy in the study eye
7. History of idiopathic or autoimmune uveitis in the study eye
8. Cataract surgery in the study eye within 90 days before Day 1
9. Aphakia in the study eye
10. Yttrium-aluminium-garnet capsulotomy in the study eye within 30 days before Day 1
11. Any other intraocular surgery in the study eye within 90 days before Day 1
12. Vitreomacular traction or epiretinal membrane in the study eye evident biomicroscopically or on optical coherence tomography that was thought to affect central vision
13. Current iris neovascularization, vitreous hemorrhage, or tractional retinal detachment in the study eye
14. Preretinal fibrosis involving the macula in the study eye
15. Structural damage to the center of the macula in the study eye that was likely to preclude improvement in best-corrected visual acuity following the resolution of macular edema, including atrophy of the retinal pigment epithelium, subretinal fibrosis or scar, significant macular ischemia, or organized hard exudates
16. Ocular inflammation including trace or above in the study eye
17. Evidence of infectious blepharitis, keratitis, scleritis, or conjunctivitis in either eye
18. Filtration surgery for glaucoma on the study eye in the past or likely to be needed in the future
19. Intraocular pressure ≥25 mmHg in the study eye
20. Myopia of a spherical equivalent prior to any possible refractive or cataract surgery of –8 or more diopters
21. Concurrent disease in the study eye, other than diabetic macular edema, that could compromise visual acuity, require medical or surgical intervention during the study period, or confound interpretation of the results (including retinal vascular occlusion, retinal detachment, macular hole, or choroidal neovascularization of any cause)
22. Only 1 functional eye even if that eye was otherwise eligible for the study
23. Ocular media of insufficient quality to obtain fundus and optical coherence tomographic images
24. Current treatment for a serious systemic infection
25. Administration of systemic antiangiogenic agents within 180 days before Day 1
26. Uncontrolled diabetes mellitus, as defined by HbA_1c_ >12%
27. Uncontrolled blood pressure (defined as systolic >160 mmHg or diastolic >95 mmHg while patient is sitting)
28. History of cerebral vascular accident and/or myocardial infarction within 180 days before Day 1
29. Renal failure requiring dialysis or renal transplant
30. History of other disease, metabolic dysfunction, physical examination finding, or clinical laboratory finding giving reasonable suspicion of a disease or condition that contraindicated the use of an investigational drug, might affect interpretation of the results of the study, or rendered the patient at high risk for treatment complications
31. Pregnant or breastfeeding women
32. Women of childbearing potential with either a positive pregnancy test result or no pregnancy test at baseline. Postmenopausal women had to be amenorrheic for at least 12 months in order not to be considered of childbearing potential
33. Sexually active men or women of childbearing potential who were unwilling to practice adequate contraception during the study are excluded (adequate contraceptive measures included stable use of oral contraceptives or other prescription pharmaceutical contraceptives for 2 or more menstrual cycles prior to screening; intrauterine device; bilateral tubal ligation; vasectomy; condom plus contraceptive sponge, foam, or jelly or diaphragm plus contraceptive sponge, foam, or jelly)
34. Allergy to fluorescein
35. Participation in an investigational study within 30 days prior to screening visit that involved treatment with any drug (excluding vitamins and minerals) or device
36. Persons with close affiliation with the investigational site, e.g., close relatives of the investigator, dependent persons, employees or students of the investigational site

**2. Investigators**

The VIVID Japan study was conducted at 17 study centers in Japan. Only investigators qualified by training and experience were selected as appropriate experts to investigate the study drug. At each center, the principal investigator was responsible for the study.

VIVID DME was conducted in 90 centers in Japan (n = 19), Europe (n = 64), and Australia (n = 7). Professor Jean-François Korobelnik (Unité Mèdicale Segment Postérieur, Service d’Ophtalmologie C.H.U. Pelligrin, Place Amèlie Raba Léon, 33000 Bordeaux, France) was the coordinating investigator.

Japanese investigators in VIVID DME and VIVID Japan were as follows: Tomohiro Otani, Masahito Ohji, Nagahisa Yoshimura, Eiichi Sato, Susumu Ishida, Motohiro Kamei, Yu Sawada, Tetsuya Baba, Yuki Morizane, Hiroshi Kunikata, Shigehiko Kitano, Taiji Sakamoto, Akito Hirakata, Atsushi Mizota, Shuichi Yamamoto, Toshinori Murata, Yuichiro Ogura, Hiroko Terasaki, Kunihiko Shiraki, Kanji Takahashi, Yasuhiro Ikeda, Takashi Kitaoka, Hitoshi Takagi, Shizuya Saika, Hiroyuki Shimada, Shigeki Yamanishi, Takashi Oyamada, Nahoko Ogata, Yasutaka Uchihori, Takafumi Koga, Yukihiro Sato, Masaki Sato, Yasutomo Tsukahara, Yoko Saigo
